# Chronic Social Defeat Stress Modulates Dendritic Spines Structural Plasticity in Adult Mouse Frontal Association Cortex

**DOI:** 10.1155/2017/6207873

**Published:** 2017-01-18

**Authors:** Yu Shu, Tonghui Xu

**Affiliations:** ^1^Britton Chance Center for Biomedical Photonics, Wuhan National Laboratory for Optoelectronics, Huazhong University of Science and Technology, Wuhan 430074, China; ^2^MoE Key Laboratory for Biomedical Photonics, Department of Biomedical Engineering, Huazhong University of Science and Technology, Wuhan 430074, China

## Abstract

Chronic stress is associated with occurrence of many mental disorders. Previous studies have shown that dendrites and spines of pyramidal neurons of the prefrontal cortex undergo drastic reorganization following chronic stress experience. So the prefrontal cortex is believed to play a key role in response of neural system to chronic stress. However, how stress induces dynamic structural changes in neural circuit of prefrontal cortex remains unknown. In the present study, we examined the effects of chronic social defeat stress on dendritic spine structural plasticity in the mouse frontal association (FrA) cortex* in vivo* using two-photon microscopy. We found that chronic stress altered spine dynamics in FrA and increased the connectivity in FrA neural circuits. We also found that the changes in spine dynamics in FrA are correlated with the deficit of sucrose preference in defeated mice. Our findings suggest that chronic stress experience leads to adaptive change in neural circuits that may be important for encoding stress experience related memory and anhedonia.

## 1. Introduction

Life stress can elicit a variety of autonomic and neuroendocrine adaptive responses for mobilizing physical resources and for enhancing survival probability. However, extreme or prolonged stress may cause cumulative and detrimental cardiovascular, metabolic, and immunosuppressive consequences [1]. Previous studies have also demonstrated that chronic stress is associated with the development of psychiatric disorders such as major depression, posttraumatic stress disorders, and anxiety [2–4]. Stress may directly cause deleterious effects on synaptic transmission and plasticity in the brain [5, 6]. Although the relationships between stress and mental illness have been established for many years, the precise mechanisms underlying how chronic stress induces mental disorders are still unclear.

Prefrontal cortex (PFC) plays a pivotal role in higher-order brain functions including memory, thought, and comprehension [7, 8]. Recent studies on structural and functional changes in the PFC under stress conditions provided valuable insight on the pathogenesis of stress-related mental disorders. Both studies examining immediate early genes (IEGs) expression in rodents and functional imaging studies in human suggest that adaptation in the prefrontal cortex is associated with the changes in emotional responses and decision making [9–12]. It has been shown that the alterations of activities in the medial and anterior regions of PFC are important for mediating stress-induced symptoms, such as depression [13, 14]. Studies using rodent stress models also showed that PFC neural circuits undergo significant structural adaptation following stress experience. For example, exposure of rodents to stress can cause changes in neuronal activity, morphology, and expression of transcriptional factors in the medial prefrontal cortex (mPFC) [15–18]. Chronic stress could induce retraction in apical dendrites and loss in dendritic spines in both layers II/III and layer V mPFC pyramidal neurons [17, 19–22]. In addition, stress decreased the number of large spines and, in contrast, increased number of small spines in layers II/III pyramidal neurons in the dorsal mPFC, suggesting homeostatic regulation of distribution of overall spine size throughout the dendrites [17]. Repeated stress also reduced volume in dorsal mPFC layer I [23] which contains apical dendrites arising from layers II/III and layer V pyramidal neurons and axons from long-range and local reciprocal connections from other cortical areas. In addition to mPFC, frontal association cortex (FrA) is also implicated in higher brain functions, and aberrant FrA activity is proposed to be involved in dementia pathology [24, 25]. Recent studies showed that the FrA is involved in associative fear learning and contributed to memory formation in associative learning [26, 27]. Structurally, FrA receives direct afferent projections from both mPFC and amygdala in rodents [26, 28, 29]. Given that FrA has rich connections with these important brain regions mediating stress responses, such as mPFC and amygdala, it is plausible that, in addition to mPFC, FrA is also involved in chronic stress-induced disorders. Therefore, stress-induced morphological changes in the FrA may have important implications for the etiology of stress-related mental disorders.

The important mechanistic insights on changes of morphology in PFC gained from previous studies are largely obtained from fixed brain tissues, which could only provide snap shot images at the end point. Given the chronic nature of stress-induced mental disorders, it is important to reveal how chronic stress affects the dynamic change of spine plasticity* in vivo* in the prefrontal cortical areas. Unfortunately, mPFC is located deep in the brain that is inaccessible for* in vivo* imaging. Unlike mPFC, FrA is located superficially and is suitable for studying synaptic plasticity* in vivo* using two-photon imaging. Dendritic spines are the postsynaptic sites where most excitatory synapses are formed, and changes in spine dynamism and morphology serve as good indicators for synaptic plasticity [30]. Dendritic spines are highly mobile and have the capacity of undergoing rapid changes in response to experience and environmental stimuli [31, 32]. For example, chronic social defeat stress (CSDS), a widely used stress manipulation by many studies [33], could produce morphological changes of neurons in many brain regions, including nucleus accumbens (NAc), amygdala, infralimbic cortices (IL), and hippocampus [34–37].

To investigate how chronic stress affects synaptic plasticity* in vivo*, we repeatedly imaged the apical dendrites of layer V pyramidal neurons in the intact FrA from mice exposed to CSDS. Neurons were identified by expression of yellow fluorescent protein (*Thy1*-YFP-H line transgenic mice) using two-photon laser scanning microscopy. We found that 10-day repeated social defeat stress resulted in a significant change in spinogenesis and survival of newly formed spines in layer V pyramidal neurons in the FrA. Our data suggest that persistence of new spines formed in the FrA after CSDS may be involved in encoding stress-related memory. The remodeling of neural circuit in FrA following exposure to CSDS also implies that stress could induce dynamic structural changes in broader frontal cortical regions besides mPFC and IL.

## 2. Materials and Methods

### 2.1. Experimental Animals

Transgenic mice sparsely labelling the layer V cortical pyramidal neurons (*Thy1*-YFP-H line on C57Bl6 background) were purchased from Jackson Laboratory and group-housed (3–5 mice per cage). 10-week-old male YFP-H line mice were used for* in vivo* imaging and behavioral tests in this study. 7-8-month-old CD-1 mice were purchased from Beijing Vitalriver (Beijing) and single housed. All the mice were offered ad libitum access to food and water and housed in a 12 h light : dark cycle (lights on at 7:00 a.m. and lights off at 7:00 p.m.). All the experimental procedures were conducted in accordance with protocols approved by the Hubei Provincial Animal Care and Use Committee and the experimental guidelines of the Animal Experimentation Ethics Committee of Huazhong University of Science and Technology.

### 2.2. Retrograde Tracing for the FrA Inputs

Cholera toxin subunit B conjugated with Alexa Fluor 647 (CTB-647) was used for retrograde tracing. Mice were anesthetized by intraperitoneal injection with 2% chloral hydrate, 10% ethyl urethane, and 1.7 mg/mL xylazine in 0.9% NaCl mixture. CTB-647 dissolved at 1 *μ*g/*μ*L concentration in saline was injected unilaterally into the superficial layer of the FrA (+2.58 bregma, +1.0 midline, and +0.08 mm ventral from dura) with a pulled glass electrode. The injection was conducted at a rate of 2.5 nL/min. Three days after the injection, mice were perfused with 4% paraformaldehyde (PFA) and brains were postfixed in 4% PFA for additional 8 hours. Brains were then sectioned with a vibratome at 50 *μ*m thickness. Confocal images were acquired by a confocal microscope (Zeiss LSM 780, 20X, NA 1.0, WD 1.8 mm).

### 2.3. Surgical Procedure for a Chronic Cranial Window

A chronic cranial window (open skull window) was surgically implanted on the* Thy1*-YFP-H line mice following the protocol described previously by Holtmaat et al. [38]. Briefly, experimental mice were anesthetized with 2% chloral hydrate, 10% ethyl urethane, and 1.7 mg/mL xylazine in 0.9% NaCl mixture (0.09 mL/10 g body weight, i.p.). Dexamethasone (0.1 mL at 0.8 mg/mL) was administered subcutaneously to prevent cerebral edema. The head of the experimental animal was stabilized with a stereotaxic frame. After removing a flap of scalp to expose the skull surface, a small drop of lidocaine (1%) and epinephrine (1 : 10^5^) mixture was applied directly onto the skull to minimize bleeding of the skin and skull. A circular groove in the skull above the FrA was thinned by a high-speed dental drill, leaving an island of skull (diameter ~ 3 mm) intact from the center. Drilling was slow and cool saline was applied periodically onto the skull to avoid heating. When the island of skull was loosened in response to a light touch by the drill head, the skull was removed and then replaced by a custom-made circular cover-glass (#1 thickness). A thin layer of cyanoacrylate was applied to the exposed skull surface and the edge of cover-glass to seal off the exterior. The cranial window was completed by applying the dental resin to cover the cover-glass edge, exposed skull, and wound margins. Additionally, a titanium bar with threaded holes was attached to skull posterior to the cranial window for head fixation during subsequent imaging sessions. The experimental mice were housed in their home cage for recovery for 14 days.

### 2.4. *In Vivo* Two-Photon Imaging

Experimental mice were anesthetized with isoflurane (3% for induction and 0.5~1% for maintenance) and placed under the two-photon laser scanning microscope with head immobilized on a custom-made head-holder through the titanium bar. Image stacks of apical dendritic segments of the FrA layer V pyramidal neurons 10–100 *μ*m below the cortical surface were acquired through the cranial window using an Olympus microscope (25X water-immersion objective, NA 1.05) with a Mai Tai Ti:sapphire laser tuned to 925 nm (output optical power < 40 mW). High-magnification images with a step size of 0.7 *μ*m were acquired for quantification of dendritic spines. A low-magnification image of the dendritic structures in the region of interest with a step size of 2.0 *μ*m and a CCD picture of the brain vasculature patterns in the same area were taken for reference images for identifying the locations for subsequent reimaging sessions. After the imaging session, the titanium bar was detached from the head-holder and the experimental animals were returned to their home cage until next imaging session. The method of* in vivo* two-photon imaging was applied identically to the FrA and the motor cortex. The imaged regions were determined according to the stereotactic coordinates. The location of the FrA for imaging was 2.58 mm anterior to the bregma and 1.0 mm lateral from the midline. The location of motor cortex for imaging was 1.3 mm anterior to the bregma and 1.2 mm lateral from the midline. The number of dendritic spines analyzed under various conditions is summarized in Supplementary Table  1 in Supplementary Material available online at https://doi.org/10.1155/2017/6207873.

### 2.5. Spine Identification and Analysis of Spine Dynamics

All analysis of spine dynamics was done manually using ImageJ software, blind to experimental conditions. The same dendritic segments from three-dimensional image stacks with high image quality were used for analysis to ensure that tissue rotation or movements between imaging sessions did not affect identification of the protrusions. Individual dendritic protrusions (length greater than one-third of the dendritic shaft diameter) were tracked manually along dendrites. Filopodia were identified as long thin structures with head diameter/neck diameter < 1.2 and length/neck diameter > 3. The other protrusions were classified as spines.

Spine dynamics, such as elimination and formation, were determined by comparing images collected at two time points. A stable spine was considered as such if it was within 0.7 mm of its expected positions between two images, on the basis of its spatial relationship to adjacent landmarks and/or its position relative to adjacent spines. An eliminated spine was the one that was present in the initial image but not the second image. A newly formed spine was the one that was absent in the initial image and then appeared in the second image. Percentages of stable, eliminated, and formed spines were all normalized to the initial image. Percentage changes in the total spine number between sessions were relative to the total spine number in the first image and calculated as percentage of formation minus percentage of elimination measured over that session.

### 2.6. Chronic Social Defeat Stress

Experimental mice were given a chronic social defeat stress (CSDS) for 10 consecutive days as previously described by Golden et al. [39]. In brief, on the first day, experimental mice were introduced to a home cage of an unfamiliar CD-1 resident for 10 min and were physically attacked. The CD-1 residents were selected based on their attack latencies. When encountering experimental mice, CD-1 mice with attack latencies shorter than 60 s on consecutive 2 daily screening sessions were chosen as aggressors. After physical attack, the intruder experimental mouse was transferred to the neighboring compartment of the resident home cage separated by a perforated Plexiglas divider and housed for the remainder of the 24 hours. On the subsequent days, the experimental mice were introduced to a novel CD-1 resident home cage and suffered physical attack and subsequent sensory contact with CD-1 aggressor residents repeated daily for 10 days. After the 10th SDS session, the experimental mice were moved to standard mouse cages for housing. Control mice were housed in pairs using an identical home cage setup, each per side divided by a perforated Plexiglas divider and handled daily.

### 2.7. Social Interaction Test

**Figure 1:**
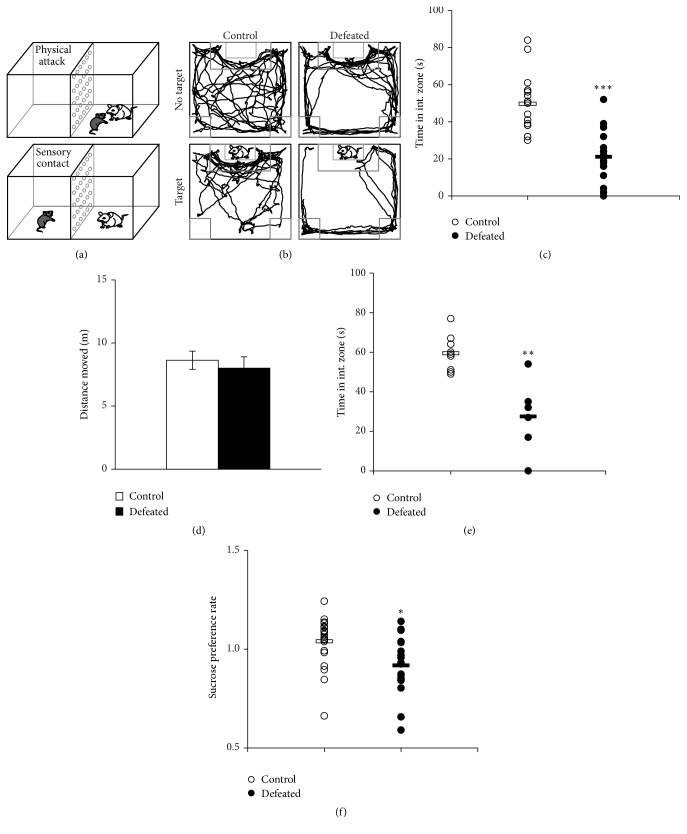
Effects of chronic social defeat stress on mice behaviors. (a) Chronic social defeat stress (CSDS) paradigm.* Thy1-*YFP-H line mice are repeatedly subjected to social defeat bouts by different stronger and aggressive CD-1 mice. Even after each 10 min defeat bout, the defeated mouse is exposed to persistent psychological stress from sensory interaction with a CD-1 mouse. (b) Social interaction test. Video-tracking data in the whole arena from control and defeated animals in the absence and presence of a social target. (c) Averaged time spent in interaction zone of control (*n* = 18) and defeated (*n* = 18) mice with a social target after a 10-day CSDS. [Student's *t*-test: ^*∗∗∗*^*P* < 0.001.] (d) Total locomotion of control (*n* = 13) and defeated (*n* = 12) mice in the whole arena during 150 s with a social target after a 10-day CSDS. [Student's *t*-test: *P* = 0.66.] (e) Averaged time spent in interaction zone of control (*n* = 9) and defeated (*n* = 6) mice with a social target after a 10-day recovery. [Student's *t*-test: ^*∗∗*^*P* < 0.01.] (f) Averaged sucrose preference rate of control (*n* = 19) and defeated (*n* = 18) mice [Student's *t*-test: ^*∗*^*P* < 0.05].

A custom-made video-tracking system was used to record the locomotion and social interaction performance of the experimental mice. The social interaction test was composed of two 150-second phases, separated by an interval of 60 seconds, either with or without a target CD-1 mouse in the interaction zone. During the first phase, the experimental mice were introduced into an open-field arena (42 × 42 cm) without a target CD-1 aggressor mouse and its moving trajectory was tracked by the custom-made video-tracking system. Immediately after the first phase, the experimental mouse was removed from the open-field arena to its home cage and a target CD-1 mouse was placed in an enclosed perforated transparent Plexiglas box that was placed into the interaction zone of the open-field arena. Then the experimental mouse was reintroduced to the open-field arena for the second testing phase. After completing the two testing sessions, both experimental mice and CD-1 mice were moved back to their home cages. The time spent in the interaction zone (an 8 cm wide corridor surrounding the box) with or without a target and the total distance travelled in the open-field arena during the two 150 s phases were recorded. Social interaction ratio was calculated as the ratio of the time experimental mouse spent in social interaction zone versus the time spent in corners (two corners of the open-field arena away from the social target; Figure 1(b)).

### 2.8. Sucrose Preference Test

Procedures for sucrose preference test were modified from Krishnan et al. [40] and Kun Li et al. In brief, the sucrose preference test consisted of two identical sessions and was performed before and after 10-day CSDS, respectively. Experimental mice were single housed and habituated to two bottles of 1% sucrose solution for 2 days. Then these bottles of  1% sucrose solution were replaced by a bottle of tap water and a bottle of  1% sucrose solution for another 2 days, during which the consumption of both sucrose solution and tap water was measured. The bottle positions were switched daily to prevent location preference. Sucrose preference was calculated as percentage of sucrose solution intake to the total intake of both sucrose solution and tap water. The sucrose preference measured before CSDS indicated the baseline preference for sucrose of the experimental mice. Sucrose preference rate was defined as the ratio of sucrose preference measured by the second testing session versus sucrose preference obtained from the first testing session.

### 2.9. Statistical Analysis

All data are presented as mean ± s.e.m. When comparing the changes in spine numbers, percentages of changes were used for the statistical analysis. Unless otherwise noted, we used two-tailed unpaired Student's *t*-tests for comparison of two groups. Two-way repeated measure ANOVA was performed to examine the percentage of total spine number during 20 days under different experimental conditions. Linear regression analysis was used to determine the relationship between spine dynamics with behaviors.

## 3. Results

### 3.1. FrA Receives Direct Inputs from mPFC and BLA

Frontal cortical regions receive convergent long-range afferent projections and local reciprocal intercortical projections. To verify the afferent projections to the FrA, we unilaterally injected retrograde tracer, CTB-647 (cholera toxin subunit B Alexa Fluor 647 conjugate), to the superficial layer of FrA in 3-month-old mice (Supplementary Figures 1A and B). Three days after injection, mice were fixed and brains were sectioned for histological analysis. We found two major clusters of CTB labelled neurons, which are located in the mPFC and the amygdala (*n* = 4 mice). Both contralateral and ipsilateral mPFC neurons were labelled, suggesting that neurons from both sides of mPFC directly project to the FrA (Supplementary Figure 1C). CTB labelled neurons in amygdala are also enriched bilaterally, particularly in the basolateral nucleus of amygdala (BLA), suggesting that FrA also receives projections from BLA bilaterally (Supplementary Figure 1D). These data confirm that FrA receives projections from mPFC and amygdala, which are known to be involved in stress and fear-related behaviors [26].

### 3.2. CSDS Induces Durable Depressant-Like Behavioral Effects

To study the effects of chronic stress on the neural plasticity in FrA, we used a social defeat behavior paradigm that significantly inhibits the motivation for social interactions in rodents [39].* Thy1*-YFP-H line mice were subjected to social defeat stress (SDS) once a day for ten days; each daily SDS episode consists of one bout of social defeat by an aggressive CD-1 mouse (aggressor) followed by continuous sensory contact with their aggressor for the rest of the day (Figure 1(a); also see methods). After ten days of CSDS, we measured social interaction behavior with an unfamiliar CD-1 mouse enclosed in a perforated transparent Plexiglas cage using a video-tracking system (Figure 1(b)). Naive control mice spent more time in the interaction zone when an unfamiliar mouse was placed in the enclosed target area than those measured with no mouse present, suggesting that the control mice tend to socially interact with an unfamiliar mouse. After experiencing ten-day SDS, animals (defeated mice) tend to display social avoidance behavior, spending less time in interaction zone near the aggressor present in the perforated cage (Figure 1(c)) [33]. CSDS does not affect basic locomotor function. There was no difference in total movement between control and defeated mice measured by the open-field behavior test (Figure 1(d)), suggesting that the decrease in time spent in target zone seen in defeated mice is not caused by any defect in basic locomotion. The social avoidance behavior could still be measured in defeated mice 10 days after recovery in their home cage, suggesting CSDS leaves a fear memory in defeated mice (Figure 1(e)). Previous studies showed that defeated mice display depression-like behavior, such as anhedonia. We performed the sucrose drinking test in control and defeated mice as well and found a significant decrease in sucrose preference in defeated mice compared to control mice (Figure 1(f)), which confirmed that the defeated mice after CSDS developed depression-like behaviors.

### 3.3. CSDS Changes Dendritic Spine Dynamics of Layer V Pyramidal Neurons in FrA

**Figure 2:**
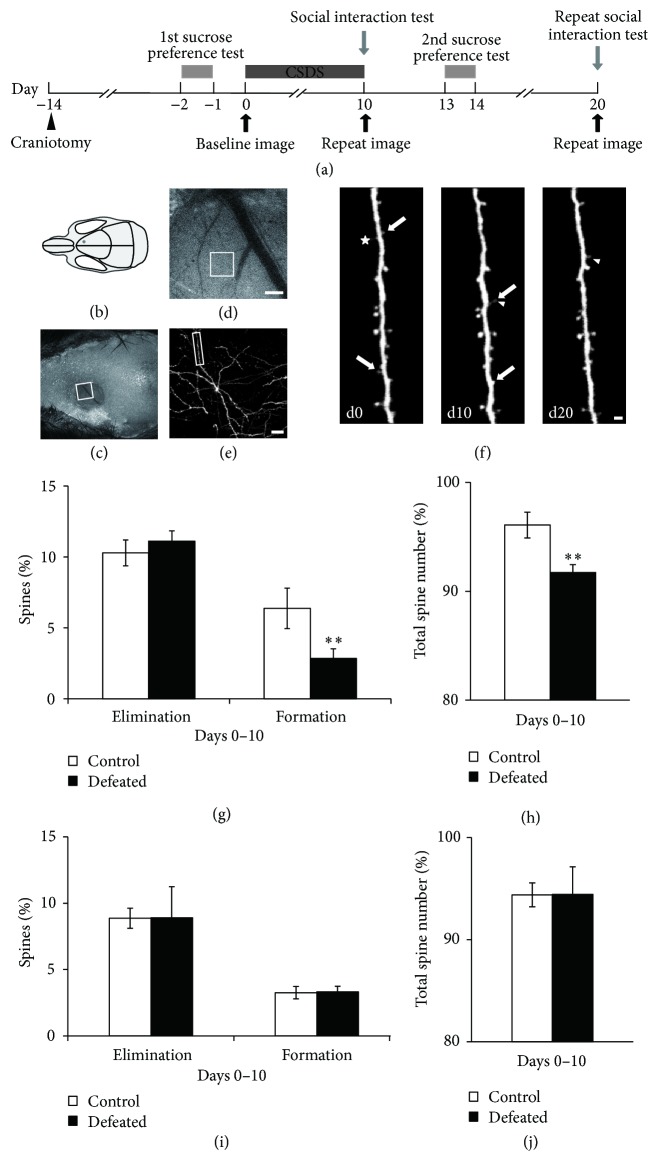
Two-photon imaging shows the altered dynamics of FrA spine in defeated mice. (a) Timeline of surgery, treatment, behavior test, and imaging. (b) Delineation of an open surgery site in mouse skull. Gray spot indicates the region where a little patch of skull over the FrA is replaced with the chronic cranial window. (c) A CCD photo of the cranial window over the FrA. Top of the skull and edge of the glass window have been covered by dental cement for fixing window and sealing off the exterior. White square indicates the interested region, which is visualized and expanded in (d) to reveal vasculature below the cranial window. (e) Two-photon image of dendritic branches and spines in the region indicated in the white box in (d). (f) Repeated imaging for the same dendritic branch segment indicated in the white rectangle in (e) at days 0, 10, and 20. Arrows and arrowheads indicate eliminated and newly formed spines, respectively. Asterisk marks dendritic filopodia. Scale bars represent 200 *μ*m (d), 20 *μ*m (e), or 2 *μ*m (f). (g) Percentage of spine elimination and formation over 10 days for control (*n* = 12) and defeated (*n* = 13) mice [Student's *t*-test: ^*∗∗*^*P* < 0.01]. (h) Percentage of total spine number in FrA of control (*n* = 12) and defeated (*n* = 13) mice after 10-day CSDS [Student's *t*-test: ^*∗∗*^*P* < 0.01]. (i) Percentage of spine elimination and formation in motor cortex over 10 days under control (*n* = 5) and CSDS (*n* = 5) conditions. (j) Percentage of total spine number in motor cortex that remain stable in control (*n* = 5) and defeated (*n* = 5) mice after 10-day CSDS.

To investigate whether CSDS modulates synaptic plasticity in FrA in defeated mice, we used two-photon microscopy to examine both formation and elimination of postsynaptic dendritic spines of layer V pyramidal neurons in FrA* in vivo*. The detailed timeline is shown in Figure 2(a). First, we performed the open skull surgery on ~10-week-old adult* Thy1*-YFP-H line mice. After two weeks of recovery from surgery, mice were repeatedly imaged before and after the CSDS experience (Figure 2(a)). Imaged regions were guided by the stereotactic coordinates; all imaged dendrites resided in the FrA (Figure 2(b)). Same dendrites were identified by using blood vessels as road map in different imaging sessions (Figures 2(c)–2(e)). By comparing the images taken before and after the CSDS in the superficial layers of the FrA, dendritic spines were identified as newly formed (arrowheads), eliminated (arrows), filopodia (asterisk), or stable (Figure 2(f)). We found that spine formation but not elimination was significantly impaired in defeated mice compared to control mice that did not experience CSDS (Figure 2(g), Supplementary Figure 2A, spine elimination: control: 10.4 ± 0.9%; *n* = 12; defeated: 11.1 ± 0.7%, *n* = 13; *P* = 0.56, Student's *t*-test; spine formation: control: 6.2 ± 1.0%; *n* = 12; defeated: 2.8 ± 0.5%, *n* = 13; *P* = 0.006, Student's *t*-test).

Decrease in spine formation and no change in spine elimination resulted in a significant but mild decrease in total spine number in defeated mice compared to that in the control mice (Figure 2(h), control mice: 95.8 ± 1.2%, *n* = 12; defeated mice: 91.7 ± 0.7%, *n* = 13; *P* = 0.007, Student's *t*-test). The change in spine dynamics found in the defeated mice was not companied by a global change throughout all cortical regions. We did not observe any change in spine formation or elimination in layer V pyramidal neurons in the motor cortex of defeated mice (Figures 2(i)–2(j), Supplementary Figure 2B, elimination and formation for control mice: 8.9 ± 0.8% and 3.3 ± 0.5%, *n* = 5; defeated mice: 8.9 ± 1.4% and 3.3 ± 0.4%, *n* = 5; *P* = 0.99 and *P* = 0.92, Student's *t*-test).

### 3.4. Enhanced Stabilization of Newly Formed Spines in Defeated Mice

**Figure 3:**
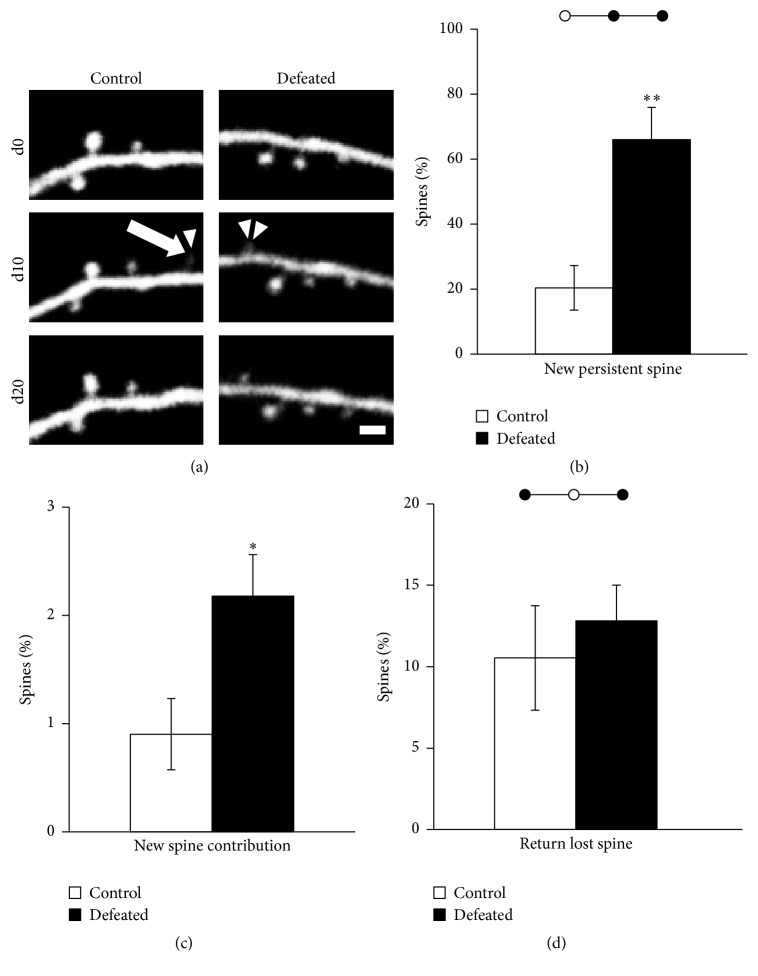
Enhanced persistence of newly formed spines in defeated mice. (a) Examples of a newly formed spine of a control (left) and a defeated (right) mouse with different fate. The defeated mouse experienced CSDS during day 0–day 10 and then recovery period during day 10–day 20. Arrowhead and arrow indicate the unstable newly formed spine which formed and then disappeared. Double arrowhead indicates the stable newly formed spine which formed and then stayed. Scale bars = 2 *μ*m. (b) Percentage of new persistent spines at day 20 in control (*n* = 9) and defeated (*n* = 10) mice [Student's *t*-test: ^*∗∗*^*P* < 0.01]. (c) Ratio of persistent new spines which formed during day 0–day 10 at day 20 in control (*n* = 9) and defeated (*n* = 10) mice [Student's *t*-test: ^*∗*^*P* < 0.05]. (d) Ratio of spines lost in day 0–day 10 and then returned at day 20 in control (*n* = 9) and defeated (*n* = 12) mice.

Because, after experiencing CSDS, defeated mice developed an enduring depressive-like behavior (Figure 1(e)), we then test whether CSDS led to any long-lasting rewiring of neural circuit. To test this, we housed defeated mice in home cage for another 10 days for recovery and reimaged the same dendritic structures to ask whether newly formed spines were retained after recovery. In control mice that did not experience CSDS, ~20% of newly formed spines were stabilized after 10 days of recovery (Figures 3(a) and 3(b), control mice: 20.4 ± 6.9%, *n* = 9), which is similar to that previously reported in other brain regions in adult mice [41]. Interestingly, we found that although spine formation was reduced by CSDS in the FrA of defeated mice, significantly higher percentage of newly formed spines during 10-day CSDS were maintained stable after 10-day recovery compared to that of the control (Figures 3(a) and 3(b), control mice: 20.4 ± 6.9%, *n* = 9; defeated mice: 66.0 ± 9.9%, *n* = 10; *P* = 0.002, Student's *t*-test).

Because there was a significant decrease in rate of spine formation but a higher rate of survival of newly formed spines, we asked what the net contribution of newly formed spines after CSDS and recovery was. To address this question, we calculated the percentage of stabilized new spines among the total spine numbers at the end of recovery period (day 20). We found that even though the spine formation was significantly reduced, stabilized newly formed spine contributed much higher percentage towards the total spine population in defeated mice than that in the control mice (Figure 3(c), control mice: 0.9 ± 0.3%, *n* = 9; defeated mice: 2.2 ± 0.4%, *n* = 10; *P* = 0.019, Student's *t*-test). In addition, we also traced the fate of spines that were eliminated during CSDS and calculated the percentage of eliminated spines reformed at the same dendritic locations to ask what percentage of eliminated synaptic contacts were reestablished after recovery. We found no difference in rate of reformed spines after elimination between control and defeated mice (Figure 3(d), control mice: 10.5 ± 3.2%, *n* = 9; defeated mice: 12.8 ± 2.2%, *n* = 12; *P* = 0.54, Student's *t*-test). Together, these data suggest that CSDS induced reorganization in synaptic connections in the FrA neural circuits, and CSDS left enduring synaptic trace by enhancing stabilization of newly formed spines in defeated mice.

### 3.5. The Spine Dynamics Is More Stable during Recovery Period in Defeated Mice

**Figure 4:**
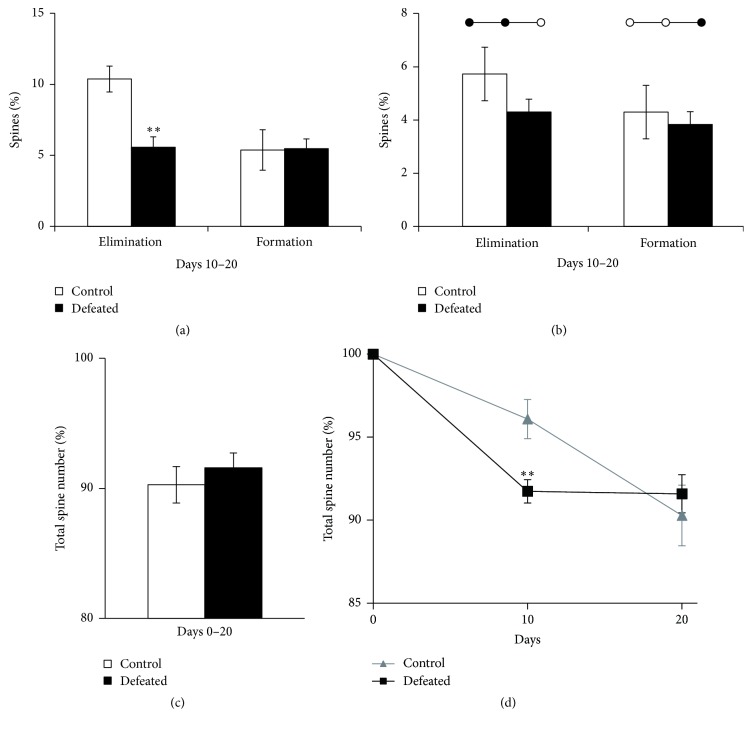
Turnover of FrA spines in control and defeated mice during recovery period. (a) Percentage of eliminated and formed spines over days 10–20 in control (*n* = 9) and defeated (*n* = 12) mice [Student's *t*-test: ^*∗∗*^*P* < 0.01]. (b) Percentage of net spine elimination and formation over days 10–20 in control (*n* = 9) and defeated (*n* = 12) mice. (c) Percentage of total spine number in FrA that remain stable at day 20 in control (*n* = 9) and defeated (*n* = 12) mice. (d) Change in total spine number during 20 days in the control (*n* = 9) and the defeated mice (*n* = 12). The defeated mice experienced CSDS during day 0–day 10 and then recovery period during day 10–day 20. [Two-way repeated measure ANOVA: time *F*(1,19) = 10.57, *P* = 0.004. CSDS *F*(1,19) = 0.444, *P* = 0.51. Time × CSDS interaction *F*(1,19) = 9.90, *P* = 0.005. Student's *t*-test: ^*∗∗*^*P* < 0.01 for control versus defeated mice on day 10.]

To examine whether 10-day CSDS experience could continue to modulate synaptic plasticity in the FrA even after exposure to stress, we studied the spine dynamics during the 10-day recovery when the control mice and defeated mice were both housed in identical home cages (day 10–day 20). Interestingly, the spine elimination rate in defeated mice was decreased compared to the control mice during recovery (Figure 4(a), spine elimination: control: 10.1 ± 1.4%, *n* = 9, defeated mice: 5.6 ± 0.7%, *n* = 12, *P* = 0.009, Student's *t*-test). In contrast, the spine formation rates did not differ between control and defeated mice (Figure 4(a), spine formation: control: 5.6 ± 1.1%, *n* = 9, defeated mice: 5.6 ± 0.7%, *n* = 12, *P* = 0.90, Student's *t*-test).

Two different scenarios could potentially cause the decrease in spine elimination rate during day 10–day 20 : increase of survival rate of new spines formed during day 0–day 10 or/and decrease in elimination rate of preexisting spines. As mentioned above, our data have shown the increase of survival rate of newly formed spines in defeated FrA. So, next we examined the elimination rate of preexisting spines during recovery, excluding the newly formed spines during 10-day CSDS. We found that there was no difference in elimination rate of the preexisting spines between control and defeated mice (Figure 4(b), control mice: 5.6 ± 1.0%, *n* = 9; defeated mice: 4.3 ± 0.5%, *n* = 12; *P* = 0.22, Student's *t*-test). In addition, we examined the formation rate during recovery excluding the returned spines which were eliminated during the first 10 days; that is, we only analyzed the new spines that appeared for the first time on last imaging day (day 20). Again, the formation rate of new spines appeared on the image was not significantly different in defeated mice compared to control (Figure 4(b), control mice: 4.6 ± 1.0%, *n* = 9; defeated mice: 3.8 ± 0.4%, *n* = 12; *P* = 0.46, Student's *t*-test). The total spine number at the end of recovery period (day 20) was not significantly different between control and defeated mice (Figure 4(c), control mice: 90.5 ± 1.8%, *n* = 9; defeated mice: 91.6 ± 1.1%, *n* = 12; *P* = 0.59). Although 10-day CSDS caused a significant decrease in total spine number in the defeated mice, the total spine number ultimately returned to the same level as control mice after 10-day recovery (Figure 4(d)), because the spine dynamics in defeated mice were more stable during recovery after CSDS.

### 3.6. Correlation between Spine Dynamics and CSDS Induced Depression Behaviors

**Figure 5:**
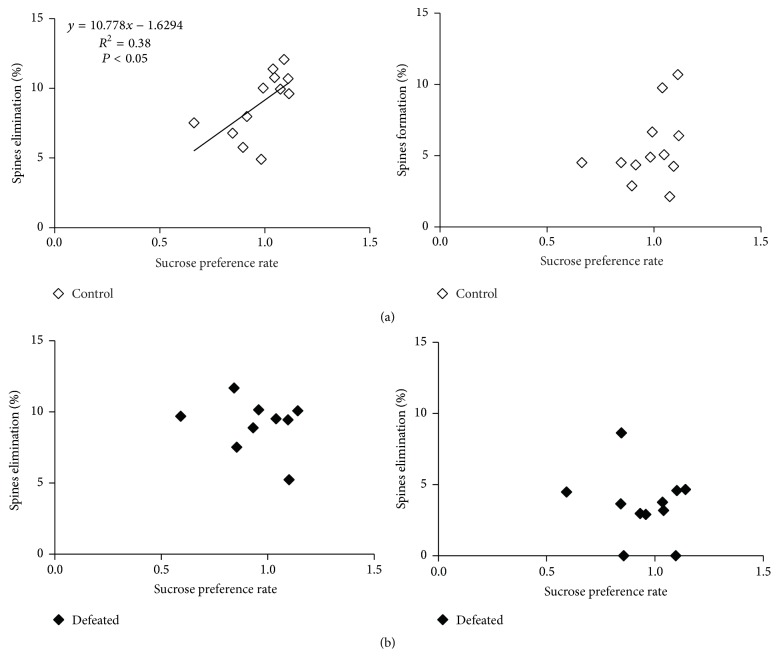
CSDS changed correlations between sucrose preference and spine dynamics in FrA. (a) Percentage of spine elimination rather than formation of control mice during day 0–day 10 was linearly correlated with sucrose preference rate (*n* = 12; linear regression analysis: elimination: *R*^2^ = 0.38, *P* = 0.032 and formation: *R*^2^ = 0.13, *P* = 0.26). (b) Percentage of spine elimination and formation of defeated mice during day 0–day 10 were both not correlated with sucrose preference rate (*n* = 11; linear regression analysis: elimination: *R*^2^ = 0.02, *P* = 0.68 and formation: *R*^2^ = 0.04, *P* = 0.58).

Changes in spine dynamics have been proposed to be one of the important mechanisms for encoding experience-dependent long-lasting memory [42]. In our study, we found that CSDS experience caused development of depression-like behaviors in defeated mice, such as social avoidance and anhedonia, that was long lasting even after 10-day recovery without continued stress. Meanwhile, in the defeated mice, CSDS induced reorganization of neural circuits in the FrA by (1) decreasing rate of the spine formation (Figure 2(g)); (2) increasing the stabilization of newly formed spines induced by CSDS (Figure 3(b)); and (3) the enhanced stabilization of synaptic connections during recovery from CSDS (Figure 3(c)). To examine whether the changes in spine dynamics were correlated with changes in depression-like behaviors, we plotted the rate of spine elimination and spine formation with that of both sucrose preference and social interaction ratio. Surprisingly, we found that the percentage of eliminated spines, but not newly formed spines, in the control mice was linearly correlated with the sucrose preference rate (Figure 5(a)). The mice with higher sucrose preference rate tend to have higher rates of spine elimination. However, such correlation was disrupted in the defeated mice (Figure 5(b)). We did not find any correlation between spine dynamics and social interaction ratio in control or defeated mice (Supplementary Figure 3). These data suggest that spine dynamics in the FrA is correlated with depression-like behaviors, and CSDS could disrupt the correlation between spine elimination and sucrose preference.

## 4. Discussion

Repeated stress has been demonstrated to play a critical role in the etiology of numerous psychiatric illnesses and induce profound morphological changes in the PFC [43]. We adopted commonly used repeated social defeat mouse model to study the effect of chronic stress on frontal cortex. Previous studies showed that chronic exposures to 10-day SDS could induce robust depression-like phenotypes, such as anxiety, anhedonia, and social avoidance behaviors in rodents [39, 44]. Compared to other environmental stressors, CSDS is unique that it can continuously activate the hypothalamic-pituitary-adrenal axis [45]. In addition, CSDS induced social avoidance can be treated and improved by chronic antidepressant treatments [33, 46]. Therefore, this model is suitable for studying chronic stress-induced depression-like behaviors. Consistent with previous studies, we found that CSDS led to the development of long-lasting social avoidance and reduced sucrose preference but no significant changes in open-field movements [39]. In the present study, we, for the first time, studied the dynamic changes of dendritic spines in the FrA of mice experiencing chronic exposure to SDS using* in vivo* two-photon microscopy. We found that ten-day continuous exposure to CSDS induced a significant decrease in rate of spine formation while it had no effect on spine elimination rate in defeated mice, which led to a decline in the total spine number in FrA. Afterward, the survival rate of newly formed spines was elevated in defeated mice. But, given that newly formed spines are more stable in defeated mice, the contribution of newly formed spines to total spine population is much higher in defeated mice compared to the control mice. Together, these results demonstrated that chronic stress could induce significant rewiring of neural circuits in the FrA of adult mice.

Progress has been made towards understanding the relationship between stress-related impairment of behavior and stress-induced structural alterations in the projection neurons within the rodent PFC. However, previous studies on stress-related remodeling in neuronal structures in the PFC were obtained primarily from fixed cortical slices tissues and were focused on examining the net changes of synapse density and morphology. Given the chronic nature of stress-related mental disorders, it is critical to determine how synaptic connections are remodeled during stress and following the recovery from stress experience. Therefore, a longitudinal study of the same dendritic structures at different time points is desired. Two-photon* in vivo* imaging on sparsely fluorescence-labelled neurons has been widely used to observe and determine the dynamic changes of the dendritic spines in superficial cortical regions. Such approach is ideal for studying the experience-dependent structural plasticity. Unfortunately, both medial and anterior frontal cortices, which are involved in stress responses, are located deep in the brain that it is not accessible for repeated* in vivo* imaging. Instead, the FrA is accessible, which allowed us to study dynamic changes in dendritic spine in animals exposed to chronic stress experience. In our study, we first confirmed that the FrA receives afferent projections from both the mPFC and the basolateral amygdala (BLA), which have been previously shown to mediate stress and fear responses. The mPFC is responsible for mediating the inhibition of negative sentiment [47, 48], and the amygdala plays important role in mediating the processing and formatting memory of negative stimuli [49, 50]. The convergent afferent projections from the mPFC and amygdala to the FrA suggest that the FrA may be important for integrating this information and directly participate in stress response.

Our study indeed revealed that the FrA is involved in synaptic plasticity following CSDS experience. We showed that the spine formation in the FrA of the defeated mice is modulated by the CSDS. The rate of spine formation is decreased; meanwhile, the survival rate of the newly formed spines during CSDS is promoted, which result in larger contribution of newly formed spines towards total spine population. These data suggest that more stress-induced new synaptic connections are integrated into the FrA neural circuits in defeated animals. It is worth noting that enhanced retention of newly formed spines is in parallel with the maintenance of social avoidance, suggesting these newly formed spines may be the memory trace induced by CSDS experience reminiscent of the selective stabilization of newly formed spines induced by motor skill training for encoding long-lasting motor memory [41]. There is a growing evidence suggesting that the long-term stabilization of experience-induced synaptic connections is a potential structural basis for memory preservation. Temporal relationship between the retention of newly formed spines and the maintenance of social avoidance behavior supports this hypothesis. In addition, recent findings suggested that the FrA is engaged in integration of external stimuli and contributes to associative learning and memory formation. For example, convergent activities from contextual stimuli and shock triggered rewiring of the FrA neural circuits [26], and protein synthesis in the FrA is necessary for the formation of associative memory [27]. Our work suggests that the FrA may be another locus encoding of chronic stress experience. Our findings that more newly formed spine are integrated into the FrA circuitry following CSDS exposure may provide a new framework for studying cellular mechanisms underlying prolonged effects of stress and fear on synaptic plasticity and depression-like behaviors.

We also found that there are positive correlations between the changes of synaptic dynamics and the stress-induced depression-like behavior. In the control mice, the sucrose preference rate is significantly correlated with the spine elimination rate (*P* < 0.05). However, this correlation is disrupted in defeated mice following CSDS, which suggests a relationship between anhedonia and abnormity of spine elimination in the FrA. The loss of correlation between the sucrose preference and the spine pruning observed in defeated mice shows a type of spine dynamic deficit induced by chronic stress.

FrA receives wide afferents from the whole brain, including the other parts of PFC, motor cortex, amygdala, thalamus, and hippocampus [28, 29], showing the region is implicated in various high brain functions, such as motor control, emotion, and memory. It has been reported that FrA is involved in associative learning and memory formation [26, 27], and abnormal activity of FrA may play a role in dementia pathology [24, 25]. However, so far there is few study about the response or adaptive changes of FrA neural circuit to the stress. The changes of spine dynamics happened in animals after exposure to CSDS, especially more new connections forming and then retention, at the first time show that chronic stress induces the remodeling of neural circuit in FrA. This remodeling may underlie the CSDS-related memory formation. Clinically, refractory and easy recurrence are the main clinic features for curing stress-related disorders such as depression and should be correlated with maintaining of stress experience memory. Our work suggests that FrA along with mPFC and amygdala should be concerned/given more attentions in exploring new treat strategy for stress-related mental disorders treatment.

In summary, the present study revealed the dynamic changes in dendritic spines in the FrA following the chronic social defeat stress experience. Our data showed that the FrA is also influenced by chronic stress, which suggests that chronic stress may impact broader frontal cortical regions besides previously identified mPFC and IL regions. Investigation of molecular and cellular mechanisms in the neural circuits of the frontal cortices involved in stress-related aberrant behaviors may facilitate a more comprehensive understanding of how chronic stress leads to mental disorders. Future study investigating the causation between rewiring of the FrA neural circuit and depression-like behaviors would shed further light on the mechanism of occurrence and persistence of the depression and potentially aid the development of therapeutic interventions for depression. Our study reveals a dynamic change in synaptic structures in the FrA in response to chronic stress experience, suggesting that the abnormal synaptic plasticity is involved in the development of the long-lasting depression-like behaviors after chronic stress.

## Supplementary Material

Supplementary figure 1: Direct inputs to FrA from mPFC and amygdala. Supplementary figure 2: Examples for repeated two-photon images of FrA and motor cortex. Supplementary figure 3: Relationship of social performance and spine dynamics in FrA. Supplementary table: Spine dynamics under different conditions.
