# Supplementary material for: Chronic Social Defeat Stress Modulates Dendritic Spines Structural Plasticity in Adult Mouse Frontal Association Cortex
Source: Neural Plast. 2017 Jan 18;2017:6207873. doi: 10.1155/2017/6207873 (PMC5286490; doi:10.1155/2017/6207873)
Supplement: Supplementary file 1 — Supplementary figure 1: Direct inputs to FrA from mPFC and amygdala. Supplementary figure 2: Examples for repeated two-photon images of FrA and motor cortex. Supplementary figure 3: Relationship of social performance and spine dynamics in FrA. Supplementary table: Spine dynamics under different conditions. [file 6207873.f1.pdf]

Chronic Social Defeat Stress Modulates Dendritic Spines Structural Plasticity in Adult Mouse Frontal Association Cortex

Yu Shu[1,2], Tonghui Xu[1,2]

[1]Britton Chance Center for Biomedical Photonics, Wuhan National Laboratory for Optoelectronics-Huazhong University of Science and Technology, Wuhan 430074, China, [2]MoE Key Laboratory for Biomedical Photonics, Department of Biomedical Engineering, Huazhong University of Science and Technology, Wuhan 430074, China.

Correspondence: Dr. Tonghui Xu (xutonghui@hust.edu.cn) Britton Chance Center for Biomedical Photonics, Wuhan National Laboratory for Optoelectronics-Huazhong University of Science and Technology, MoE Key Laboratory for Biomedical Photonics, Department of Biomedical Engineering, Huazhong University of Science and Technology, Wuhan 430, China

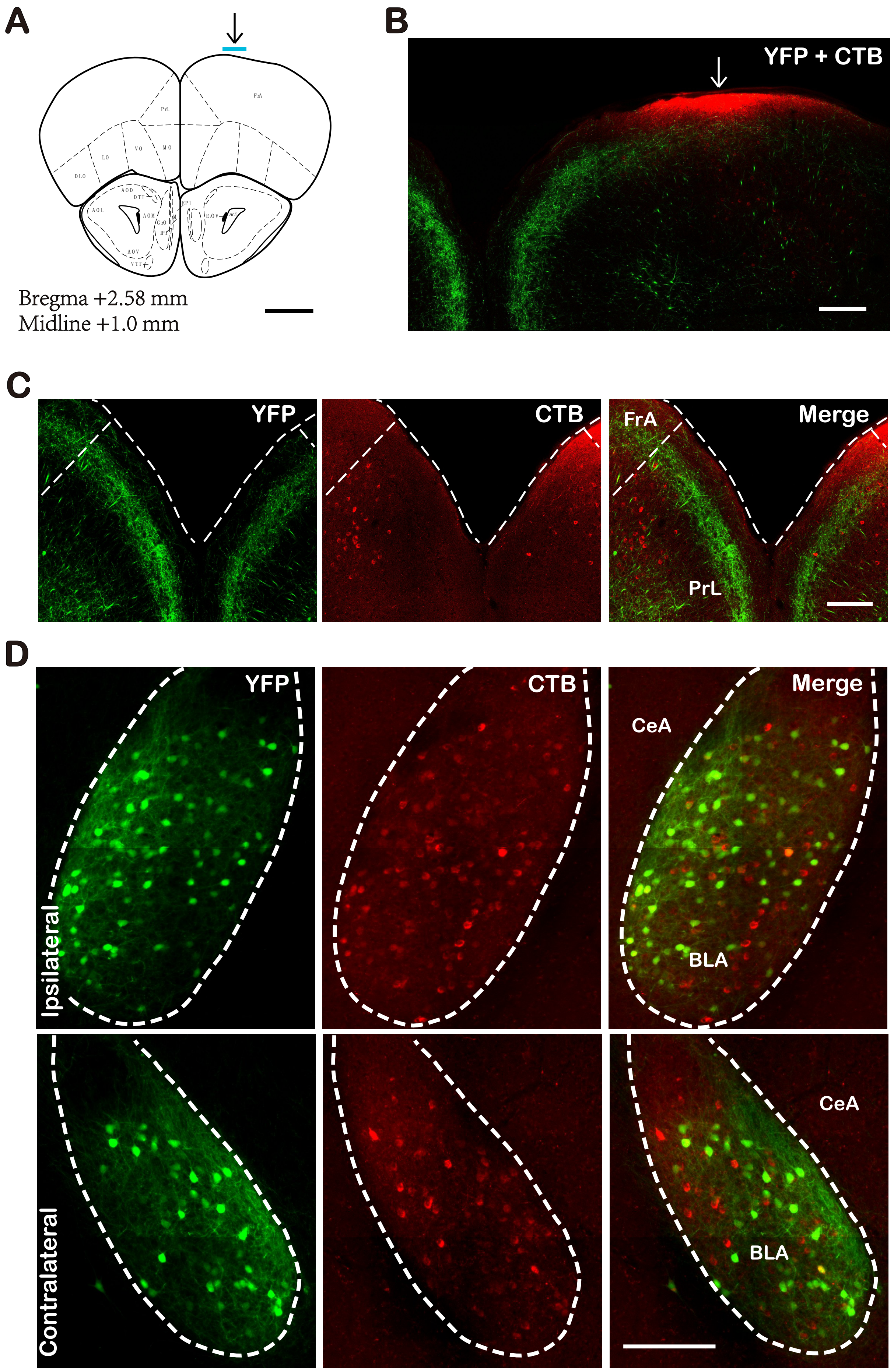
A
B
YFP + CTB
FrA
PrL
LO
VO
MO
DLO
Bregma +2.58 mm
Midline +1.0 mm
C
YFP
CTB
FrA
Merge
PrL
D
YFP
CTB
Merge
CeA
BLA
Ipsilateral
Contralateral
CeA
BLA

Supplementary Figure 1

Direct inputs to FrA from mPFC and amygdala.

(A) Diagram of the mouse frontal association cortex (FrA) in a coronal section. Arrow indicates the injection site of cholera toxin subunit B Alexa Fluor 647 conjugate (CTB-647) into the imaging site (blue) of FrA for retrograde tracing (+2.58 mm bregma, +1.0 mm midline, +0.08 mm ventral from dura). Scale bar: 1mm. (B) Confocal image of a coronal section with the CTB-647 injection showing the neural fibers and neurons labeled with CTB-647 (red). (C) Confocal images of pyramidal neurons retrograde labeled bilaterally by CTB-647 in mPFC (bregma +2.80 mm). (D) Confocal images of neurons retrograde labeled by CTB-647 ipsilaterally (top) and contralaterally (bottom) in the basolateral nucleus of the amygdala (BLA, bregma -1.22 mm). Scale bar: 200µm (B, C, D). Mice number: n = 4.

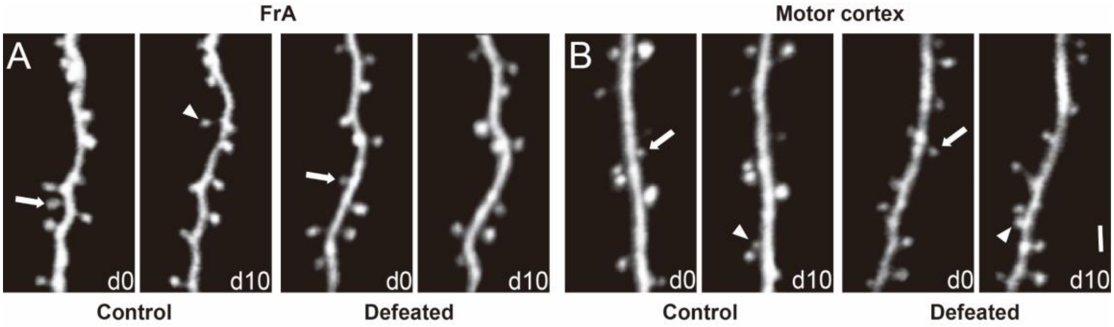

Supplementary Figure 2

Repeated two-photon imaging of the same dendritic segment and spines in FrA and motor cortex of control and defeated mice.

(A) Examples of repeated two-photon images of the same dendritic segment in the FrA of a control (left) and a defeated (right) mouse. The imaging location of FrA was 2.58 mm anterior to the bregma and 1.0 mm lateral from the midline. (B) Examples of repeated two-photon images of the same dendritic segment in the motor cortex of a control (left) and a defeated (right) mouse. The imaging location of the motor cortex was 1.3 mm anterior to the bregma and 1.2 mm lateral from the midline. Arrow and arrowhead indicates the eliminated and newly formed spine respectively. Scale bar = 2µm.

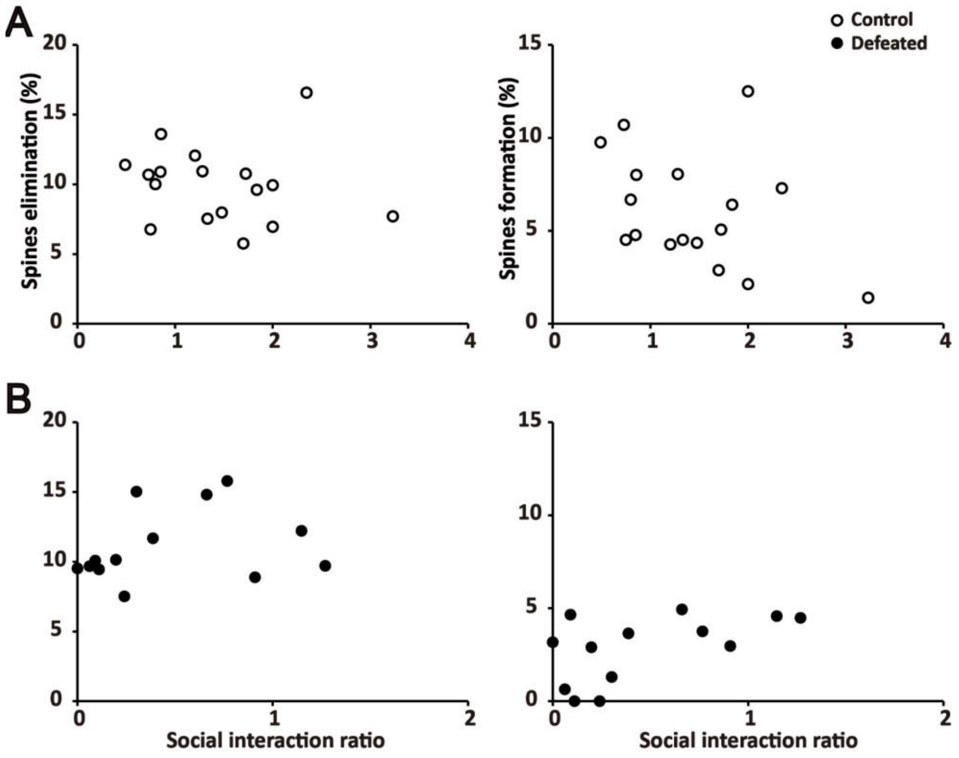

Supplementary Figure 3

No correlation was found between social interaction ratio and spine dynamics in FrA.

(A) Percentage of spine elimination and formation of control mice during day0-day10 was l was not correlated with social interaction ratio (n = 17. Linear regression analysis: Elimination: R^2 = 0.01, *P* = 0.65. Formation: R^2 = 0.14, *P* = 0.13). (B) Percentage of spine elimination and formation of defeated mice during day0-day10 were not correlated with social interaction ratio (n = 13. Linear regression analysis: Elimination: R^2 = 0.07, *P* = 0.40. Formation: R^2 = 0.31, *P* = 0.05).

Supplementary Table 1:

Imaging interval	Experimental conditions	Spine elimination (%)			Spine formation (%)			Spine density (%)			Total number of spines (n)	Total number of mice (N)
		Mean	SD	SEM	Mean	SD	SEM	Mean	SD	SEM		
Day 0-10	Control	10.4	3.0	0.9	6.2	3.3	1.0	95.8	3.9	1.2	1684	12
	Defeated	11.1	2.6	0.7	**2.8	1.8	0.5	**91.7	2.5	0.7	1793	13
Day 10-20	Control	10.1	4.0	1.4	5.6	3.1	1.1	90.5	5.2	1.8	1173	9
	Defeated	**5.6	2.4	0.7	5.5	2.2	0.6	91.6	4.0	1.1	1515	12

Supplementary Table 1:

Percentages of dendritic spines eliminated and formed under different experimental conditions over CSDS and recovery period. Images were obtained from defeated mice and their control littermates. All data points were obtained from the frontal association cortex. For the convenience of reference, both SD value and SEM value are presented. (**P<0.01 compared to controls, Student t-test.)
